# Crisis of Meaning and Subjective Well-Being: The Mediating Role of Resilience and Self-Control among Gifted Adults

**DOI:** 10.3390/bs10010015

**Published:** 2019-12-26

**Authors:** Bernadette Vötter

**Affiliations:** Institute of Psychology, University of Innsbruck, Innrain 52, 6020 Innsbruck, Austria; bernadette.voetter@gmx.net; Tel.: +43-512-507-56043

**Keywords:** crisis of meaning, meaning in life, subjective well-being, resilience, self-control, giftedness, multiple mediation analysis, intellectual giftedness, high achievers, academically high-achieving adults

## Abstract

Meaning in life is positively associated with mental and physical health, while a crisis of meaning is a painful existential state that is defined as a perceived lack of meaning. An earlier study has shown that academically high-achieving adults mostly experience existential fulfilment, while intellectually gifted adults have a disproportionally high risk of suffering from a crisis of meaning, which can weaken their potential fulfilment in life. To uncover the underlying mechanisms of how an existential crisis affects gifted adults’ mental health, this study examines the longitudinal relationship between crisis of meaning and subjective well-being via two mediators: self-control and resilience. A multiple mediation model was tested with longitudinal data (two times of measurement) of two gifted groups: intellectually gifted adults (HIQ; N = 100; 55% female) and academically high-achieving adults (HAA; N = 52; 29% female). Results suggest group differences: HIQ had higher crisis of meaning and lower self-control than the HAA. HIQ’s resilience (but not their self-control) and HAA’s self-control (but not their resilience) mediated the relationship between crisis of meaning and subjective well-being. These findings give initial insights about the distinct psychological needs of gifted adults and their different paths toward subjective well-being. These insights can be applied in future giftedness research, talent development programs, or counseling to support gifted individuals in living up to their potential. Thus, HIQ could benefit particularly from supporting their ability to cope with adversity, while HAA could benefit particularly from strengthening their willpower to modify undesired emotions, behaviors, and desires.

## 1. Introduction

Existential aspects of health have gained growing interest in academia and the general public [1,2,3,4]. Meaning in life has well-documented benefits on mental and physical health [2,5]. It can be described by two dimensions: meaningfulness and crisis of meaning [2,6]. Experiencing meaningfulness is based on a validation of one’s life as coherent, significant, directed, and belonging [7]. A positive appraisal of these components mostly occurs unconsciously, while a perceived lack of meaning in life occurs consciously and is known as a crisis of meaning [2,7].

Individuals who are suffering from a crisis of meaning often judge their lives as frustratingly empty and pointless. They feel disorientated, experience depression, and suicidal ideations [7,8]. Moreover, this existential state is linked with heightened anxiety, negative affect, and pessimism, on the one hand, and decreased resilience, motivation, life satisfaction, hope, self-regulation, and self-efficacy, on the other hand [7,8,9,10]. A crisis of meaning is often caused by critical life events (e.g., failure, illness, death, divorce, or job loss), linked with a loss of one’s sense of coherence in life, and results in questioning life’s purpose [2,7,11].

The two predominantly used categories to classify an individual as gifted are giftedness as potential and giftedness as manifested talent or achievement [12,13,14]. Individuals who represent the first category (giftedness as potential) have an outstanding high intellect (IQ ≥ 130) and are labeled as intellectually gifted [12,14,15]. Due to their high intelligence, these individuals have a high potential for success in life but merely in combination with favorable environment and socio-psychological variables [16]. Individuals who represent the second category (giftedness as manifested talent) achieved expertise or extraordinarily high performance in a certain domain (e.g., academic achievement) and are labeled as high achievers [13,14]. Academic achievement and cognitive abilities are moderately to strongly associated [17,18]. However, merely half of the variance in academic achievement can be explained by cognitive abilities [18], the rest of the variance is accounted for by other factors, such as personality, motivation, and socio-psychological variables [19,20]. This study included representatives of both categories to uncover their specific psychological group needs.

Several scholars [14,21,22] have argued that intellectually gifted and high achieving individuals have overlapping characteristics but are distinct gifted groups. In line with this assumption, recent research suggests that intellectually gifted adults have lower subjective well-being and meaningfulness than academically high-achieving adults or a control group [14]. Moreover, a cross-sectional study reveals a disproportionally high risk of suffering from a crisis of meaning among intellectually gifted adults, while the majority of the academically high-achieving adults experience a sense of meaning [23]. The reported frequency of crisis of meaning among the intellectually gifted group (15%) was almost four times higher than among the academically high-achieving group or a control group from the general population [23]. These findings emphasize that academically high-achieving adults tend to experience existential fulfilment, while intellectually gifted adults have a tendency for existential frustration. This is particularly alarming when considering that intellectually gifted adults’ meaning in life predicted their subjective well-being in a longitudinal study [24]. A broad body of studies with intellectually gifted persons suggests a similar tendency for psychosocial issues, such as a vulnerability for affective disorders and immune-related diseases [25], identity problems, compulsivity [26], a low sense of coherence [27], a fearful attachment style [28], and the perception of being different [29].

Given these findings about psychosocial issues among some gifted individuals, it remains unclear whether negative psychological conditions (i.e., crisis of meaning) influence their well-being directly or whether there are other underlying mechanisms that lead to a decrease of their well-being and potential fulfilment. Thus, I hypothesize that a crisis of meaning decreases gifted adults’ subjective well-being via a reduction of their psychosocial adjustment resources (e.g., resilience and self-control).

Resilience refers to the ability to cope with and bounce back quicker from adversity and is associated with psychological well-being [30,31,32]. Resilient individuals are better equipped to deal with critical life events, challenges, and show adaptive behavior [30,31,32,33]. However, stress, depression, anxiety, or a crisis of meaning can decrease resilience [8,34,35]. Luthar [36] suggests individuals with high intelligence have an enhanced sensitivity to stressors, which can decrease their resilience. Given the previously mentioned tendency for psychosocial issues among intellectually gifted individuals (e.g., affective disorders, a low sense of coherence, or a low sense of meaning), I propose that their resilience could be substantially afflicted when facing a crisis of meaning, which, in turn, is hypothesized to decrease their subjective well-being.

Self-control refers to the ability to override or modify inner responses and to interrupt undesired thoughts, feelings, and behavior [37,38,39]. The perception of personal control is associated with mental and physical health, psychological well-being, positive adaption, academic success, high performance, and lower reactivity to stressors [40,41,42,43,44,45,46,47]. The ability to regulate one’s responses is strongly associated with motivation, which, in turn, is linked to goal achievement and, according to goal theories, results in higher well-being [48,49,50]. A recent longitudinal study suggests that self-control strengthens the positive association between a sense of meaning and subjective well-being among intellectually gifted adults [51]. Moreover, this study emphasizes that academically high-achieving adults’ subjective well-being is strongly linked with self-control [51]. Given these suggestions and the finding that self-control deteriorates during stressful life periods [52], I propose that a crisis of meaning decreases self-control, and, in turn, subjective well-being among intellectually gifted and academically high-achieving adults.

Both resilience and self-control can support an active, self-determined way of living, which can assist gifted individuals in living up to their potential. However, critical live periods can deteriorate both resources [8,34,35,52]. Given the previously mentioned findings that have suggested group differences [14,23], I propose group differences, with intellectually gifted adults having higher crisis of meaning, lower resilience, and self-control than the academic achievers (Hypothesis 1). In addition, this study aims to reveal whether a crisis of meaning leads to a decrease in subjective well-being via a decrease in both resilience and self-control among (a) intellectually gifted and (b) academically high-achieving adults (Hypothesis 2). Moreover, I hypothesize that intellectually gifted adults might follow a different path toward subjective well-being than academically high-achieving adults (Hypothesis 3).

## 2. Methods

### 2.1. Participants

Longitudinal data of two gifted groups—intellectually gifted adults (HIQ) and academically high-achieving adults (HAA)—was collected via an online survey as part of a larger research project at two times of measurement (t1 and t2, with a time lag of four years). This longitudinal research design allowed to examine long-term effects of life meaning on mental health-related variables, i.e., underlying mediation effects of crisis of meaning on subjective well-being.

The HIQ group (N = 100) represents the previously mentioned category “giftedness as potential” [12,13,14]. Participants were recruited through the Austrian and German high IQ society Mensa. The prerequisite for membership in Mensa is a documented IQ of 130 or higher. At measurement time 1, 148 Mensa members participated in the online study and 102 of them gave their approval to be contacted for a follow-up study. Four years later, these 102 were invited via e-mail to participate in the follow-up study and 100 participated at measurement time 2 (attrition rate between t1 and t2: 32%). All subsequent analyses are based on the data of the 100 intellectually gifted adults who participated at both times of measurement. Fifty-five percent of them were female (M_age_ = 43, SD = 9). Eighty-three percent were Germans, 16% were Austrians, and 1% had another nationality.

The HAA (N = 52) group represents the previously mentioned category “giftedness as manifested talent” [12,13,14]. Prior to t1, all participants of this group had obtained a doctorate *sub auspiciis praesidentis rei publicae*, which is the highest possible academic honor a student can achieve at Ph.D. level in Austria. To qualify for this honor, all grades—starting from high school up to tertiary education—have to be excellent and the student needs to graduate with the distinction *summa cum laude* at every educational level. At measurement time 1, 92 academically high-achieving adults participated in the online study and all of them gave their approval to be contacted for a follow up study. Four years later, they were invited via e-mail to participate in a follow-up study and 52 of them participated at measurement time 2 (attrition rate between t1 and t2: 43%). All subsequent analyses are based on the data of these 52 academically high-achieving adults who participated at both times of measurement. All of them were Austrians and 29% were female (M_age_ = 57, SD = 14).

Post-hoc power analyses (G*Power) [53] were conducted to investigate the power of the study based on the sample sizes of the two groups. Results indicated adequate statistical power to detect at least medium effects for both groups (HIQ: 1 − β = 0.99; HAA: 1 − β = 0.87).

### 2.2. Measures

At measurement time 1, crisis of meaning was assessed by five items out of the Sources of Meaning and Meaning in Life Questionnaire (SoMe; German version: LeBe) [6,7]. Items were rated on a six-point Likert scale from 0 (strong disagreement) to 5 (strong agreement). Internal consistency was good for crisis of meaning (α_HIQ_ = 0.95; α_HAA_ = 0.78).

After four years, at measurement time 2, the three other variables (subjective well-being, resilience, and self-control) were assessed. Subjective well-being was measured by the WHO-5 Well-being Index (WHO-5) [54]. The five items were rated on a six-point Likert scale from 0 (at no time) to 5 (all the time). The scale measures a subjective account of well-being based on positive mood and vitality. WHO-5 demonstrated good internal consistency in this study (α_HIQ_ = 0.82; α_HAA_ = 0.80). Resilience was assessed using the short German version of the resilience scale (RS-13) [31,32]. The 13 items were rated on a seven-point Likert scale from 1 (disagree) to 7 (agree) and measured an individuals’ ability to cope with and bounce back from adversity. Possible scores range from 13 to 91 with higher scores indicating higher resilience. Internal consistency in the current study was good (α_HIQ_ = 0.81; α_HAA_ = 0.89). Self-control was measured using the short German version of the self-control scale (SCS-KD) [37,55]. The 13 items were rated on a five-point Likert scale from 1 (totally disagree) to 5 (totally agree). The scale measures the ability to override or modify one’s inner responses and the ability to interrupt undesired behaviors. SCS-KD showed good internal consistency (α_HIQ_ = 0.85; α_HAA_ = 0.86).

### 2.3. Data Analysis

**Figure 1:**
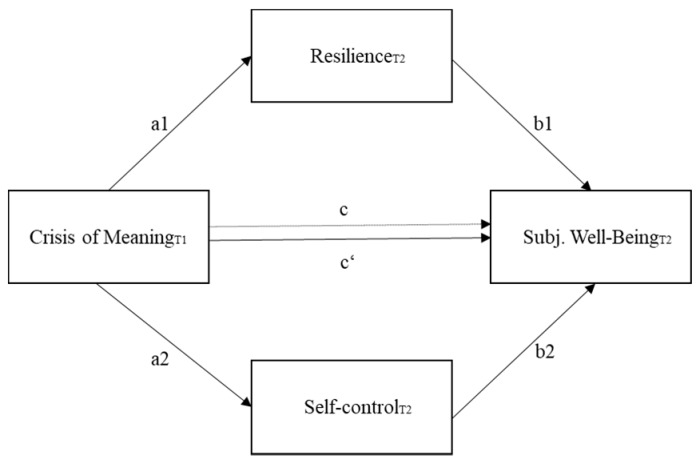
Multiple mediation model illustrating the association between crisis of meaning and subjective well-being via resilience and self-control. The arrow marked with c denotes the total effect and the arrow marked with c’ denotes the direct effect.

To test the three hypotheses, I used IBM Statistical Package for the Social Science (SPSS; version 24). First, I analyzed descriptive statistics and Cronbach’s alpha of all measures. Correlation coefficients of r = 0.10–0.29 were interpreted as a weak effect, coefficients of r = 0.30–0.49 were interpreted as a moderate effect, and coefficients of r ≥ 0.50 were interpreted as a strong effect [56]. Second, I utilized a MANCOVA to test for group differences within the observed variables. Third, I used PROCESS (Version 3.1), a macro for SPSS developed by Hayes [57] to conduct multiple mediation analyses (model number 4). Including a mediator gives a better understanding of the underlying mechanisms, which influence the association between the independent and dependent variable [58]. In addition, a multiple mediation analysis allows for the simultaneous examination of multiple mediators, whereby effects of each mediator are reported, while controlling for all other proposed mediators [57]. I tested multiple mediation models for both groups, with crisis of meaning as independent variable, subjective well-being as dependent variable, resilience and self-control as mediators (see Figure 1).

Variables were mean centered to minimize multi-collinearity. I applied a bootstrap method recommended by Preacher and Hayes [59], 5000 resampling with replacement. Bootstrapping has multiple strengths [57,59]. First, it provides reliable estimates of indirect effects. Second, it has higher power and better Type I error control than alternative mediation analyses. Third, bootstrapping produces a distribution using the observed data to estimate statistical effects and is therefore recommended for analyses with small sample sizes. Fourth, bootstrapping does not require standard normality of the sampling distribution [57,59]. As recommended by Hayes [57], the indirect effect is interpreted as significant when the upper and lower bounds of the 95% percentile confidence intervals, based on 5000 bootstrap samples, do not contain zero [57,60]. This approach anticipates that 95% of the confidence interval contains the true parameter value [57].

## 3. Results

### 3.1. Descriptive Statistics and Preliminary Analysis

Scale reliabilities, correlations, and descriptive statistics (mean and standard deviation) for all observed variables are shown in Table 1. Correlation analysis revealed strong negative associations between crisis of meaning and the other three positive psychological constructs (subjective well-being, resilience, and self-control) among the HAA. Strong positive associations were found between subjective well-being, resilience, and self-control. The pattern of correlations was similar among the HIQ group. Cronbach’s alpha was good, ranging from α = 0.78 (for crisis of meaning among HAA) to α = 0.95 (for crisis of meaning among HIQ).

To test hypothesis 1, I applied a MANCOVA to test for group differences in terms of the observed variables and included demographics (age and gender) as covariates. Results revealed a significant general effect for the group variable (Wilks-Lambda = 0.869, F (4,145) = 5.443, *p* < 0.001, η^2^ = 0.131) and age (Wilks-Lambda = 0.930, F (4,145) = 2.735, *p* = 0.031, η^2^ = 0.070). Data revealed significant intersubjective effects for crisis of meaning (F (1,148) = 10.865, *p* = 0.001, η^2^ = 0.068) and self-control (F (1,148) = 14.383, *p* < 0.001, η^2^ = 0.089), while resilience (F (1,148) = 2.029, *p* = 0.156, η^2^ = 0.014) and subjective well-being (F (1,148) = 2.471, *p* = 0.118, η^2^ = 0.016) had no intersubjective effects. Thus, HIQ showed higher crisis of meaning and lower self-control than the HAA. No group differences revealed for resilience or subjective well-being.

### 3.2. Multiple Mediation Analyses

To test hypothesis 2, I conducted multiple mediation analyses to test whether resilience and/or self-control mediate the negative relationship between crisis of meaning and subjective well-being among both gifted groups (see Figure 1). Table 2 presents the main results of the analyses. As recommended by Hayes [57], the path coefficients are reported in unstandardized form and the coefficients of the specific indirect effects of the mediators are additionally reported in completely standardized form.

Partially in line with the assumption, results indicate an indirect effect of crisis of meaning on subjective well-being via resilience, while controlling for self-control among the HIQ group. However, contrary to hypothesis 2, self-control did not mediate the association between crisis of meaning and subjective well-being among the HIQ group. Thus, two intellectually gifted individuals who differed by one unit in their experienced crisis of meaning were estimated to differ by −0.69 units in their experienced subjective well-being as a result of the tendency of those higher in crisis of meaning to experience lesser resilience, which in turn translates into a decrease in subjective well-being. The direct effect of crisis of meaning on subjective well-being was statistically significant. The overall multiple mediation model accounted for 40% of the variance in subjective well-being among the HIQ group.

As proposed and similar to the HIQ group, findings suggest an indirect effect of crisis of meaning on subjective well-being, however, via self-control among the HAA, while controlling for resilience. However, resilience did not mediate the association between crisis of meaning and subjective well-being among the HAA group. These findings partially confirm hypothesis 2. Thus, two academically high-achieving individuals who differed by one unit in their experienced crisis of meaning were estimated to differ by −1.16 units in their experienced subjective well-being as a result of the tendency of those higher in crisis of meaning to experience lesser self-control, which in turn translates into a decrease in subjective well-being. The direct effect of crisis of meaning on subjective well-being was statistically significant among the HAA group. The multiple mediation model explained 66% of the variance in subjective well-being among the HAA group.

Results confirmed hypothesis 3, which proposed group-differences with regard to the mediators: crisis of meaning decreased HIQ’s resilience and HAA’s self-control, which, in turn, decreased their subjective well-being.

## 4. Discussion

This study aimed to reveal underlying psychological mechanisms (i.e., resilience and self-control) that mediate the association between crisis of meaning and subjective well-being among gifted individuals. Several scholars [14,21,22] recommended a differentiation between various facets of giftedness. Based on this recommendation, I analyzed longitudinal data of representatives of “giftedness as potential” (HIQ group) and representatives of “giftedness as manifested achievement” (HAA group) to discover specific psychological needs of these groups.

The HIQ group reported, as expected in hypothesis 1, higher crisis of meaning and lower self-control than the HAA group. These results are in line with findings of scholars who suggested existential troubles [14,23], lower psychosocial adjustment among the HIQ [25,26,27,28,29], and group differences between HIQ and HAA [14,23].

In accordance with hypothesis 2, crisis of meaning decreased both gifted groups’ subjective well-being via a reduction of their psychological adjustment resources. Moreover, results revealed group differences with regard to the mediators in accordance with hypothesis 3. HIQ’s resilience (but not self-control) mediated the relationship between crisis of meaning and subjective well-being. Thus, their decreased ability to cope with adversity decreased their subjective well-being. Given these findings, one could assume that intellectually gifted individuals lack problem-focused coping strategies, which, in turn, leads to a decrease in their subjective well-being. These findings support Luthar’s [36] suggestion that individuals with high intelligence have an enhanced sensitivity to stressors, which, in turn, lowers their ability to bounce back from adversity.

In accordance with hypothesis 3, findings revealed contrasting results regarding the mediators among the HAA group. Self-control (but not resilience) mediated the relationship between crisis of meaning and subjective well-being. Thus, crisis of meaning diminished HAA’s capacity to delay undesired emotions, behavior or desires. This inflexibility to respond consciously to stimuli (internal or external) and the missing focus of awareness—when suffering from a lack of meaning—in turn, decreased their subjective well-being. These findings are in accordance with a recent longitudinal study that suggests a strong link between self-control and subjective well-being among academically high-achieving adults [51].

The results of this study demonstrate that a lack of meaning can reduce the beneficial effects of resilience among HIQ and self-control among HAA. Both resilience and self-control are beneficial resources with the potential to support gifted individuals in living happy and fulfilled lives. Thus, HIQ could particularly benefit from supporting their ability to cope with adversity, while HAA could particularly benefit from strengthening their willpower to modify undesired emotions, behaviors, and desires. Scholars and practitioners could apply these findings in, e.g., talent development trainings, future (interventional) studies or counseling of gifted persons. However, these are first findings and, thus, need replication studies to draw more valid conclusions. Moreover, future studies could examine whether gifted individuals can benefit from resilience trainings that enhance problem-focused coping strategies or self-regulation trainings that improve their ability to delay gratification [38,45].

In addition, results emphasize that the groups use different psychological adjustment mechanisms when facing critical life events (i.e., crisis of meaning), which endorse the assumption that these two groups have overlapping characteristics but are indeed distinct groups [14,21,22]. Given these findings, I encourage scholars to distinguish between different facets of giftedness, e.g., intellectually giftedness and high achievement in future studies.

One strength of this study is its longitudinal design, which allowed the investigation of multiple mediating associations between a lack of meaning and subjective well-being over time. The longitudinal design allows for a more accurate prediction of these associations than a cross-sectional design. Two times of measurement were sufficient to test the proposed multiple mediation models. However, future studies could consider more times of measurement, which would allow to gain more insights about certain long term effects of life meaning on gifted individuals’ well-being and mental health. A second strength is the diversity of the two gifted groups, which enabled the analysis of specific psychological needs. Despite these strengths, some limitation should be considered. First, the reported associations might not be causal but influenced by further, non-assessed, constructs. Second, the sample included two pre-selected gifted groups: (a) members of the high IQ society Mensa and (b) academic achievers who obtained their doctorate *sub auspiciis praesidentis rei publicae*, which can compromise the generalizability of the results to other (unselected) gifted individuals. In addition, most of the participants were German or Austrian, which can compromise the generalizability of the results to other nationalities. Third, the data are based on self-reports of the 152 participants. Thus, there is a risk of selective recollection of data and over- or under-reporting. However, the assessed scales have been shown to be reliable and valid measures throughout numerous studies [6,7,31,32,37,54,55].

## 5. Conclusions

The findings of this study contribute to a more comprehensive empirical understanding of meaning in life, happiness, and the role of underlying mediating mechanisms (i.e., resilience and self-control) among intellectually gifted adults and academically high-achieving adults. The revealed group differences give valuable insights about different psychological and developmental needs of gifted individuals. Raising awareness for the existential and psychological needs of gifted individuals and integrating these needs in talent development programs, counseling, and future giftedness research can support gifted individuals in reaching their best potential and living fulfilled and happy lives. Comprehensive talent development programs that combine the development of intellectual skills, existential, and psychological needs have the potential to foster gifted individuals’ growth, which, in turn has multiple benefits for the society, e.g., innovation, competitive advantage, efficiency, or progress in science [61].

## Figures and Tables

**Table 1:** Pearson correlations, means, standard deviations, and reliabilities.

Variables	1		2		3		4	
	HIQ	HAA	HIQ	HAA	HIQ	HAA	HIQ	HAA
1. CoM_T1_	(0.95)	(0.78)						
2. SWB_T2_	−0.50 **	−0.76 **	(0.82)	(0.80)				
3. Res_T2_	−0.52 **	−0.80 **	0.58 **	0.67 **	(0.81)	(0.89)		
4. SC_T2_	−0.26 *	−0.53 **	0.31 **	0.65 **	0.37 **	0.56 **	(0.85)	(0.86)
M	1.48	0.40	13.53	16.60	69.40	73.75	3.12	3.67
SD	1.38	0.65	4.41	4.21	9.38	10.60	0.65	0.60
Range	0–5	0–5	0–25	0–25	13–91	13–91	1–5	1–5

Note. Cronbach’s alpha are provided in parentheses on the diagonal. HIQ (High Intellectually Gifted Adults); HAA (Academically High-Achieving Adults); CoM (Crisis of Meaning); SWB (Subjective Well-Being); Res (Resilience); SC (Self-Control); T1 (Measurement Time 1); T2 (Measurement Time 2). ** *p* < 0.001, * *p* < 0.01.

**Table 2:** Path coefficients of the multiple mediation analyses, standard errors, and 95% percentile confidence intervals predicting subjective well-being among Intellectually Gifted Adults (N = 100) and Academically High-Achieving Adults (N = 52).

	Intellectually Gifted Adults (N = 100)				Academically High-Achieving Adults (N = 52)			
Path	Coeff.	SE	LLCI	ULCI	Coeff.	SE	LLCI	ULCI
Total effect (c)	−1.60	0.28	−2.15	−1.04	−4.90	0.59	−6.09	−3.72
Direct effect (c’)	−0.84	0.30	−1.43	−0.24	−3.59	0.92	−5.43	−1.75
a1	−3.55	0.59	−4.72	−2.39	−13.00	1.37	−15.75	−10.25
a2	−0.12	0.05	−0.22	−0.03	−0.49	0.11	−0.71	−0.27
b1	0.19	0.05	0.10	0.28	0.01	0.06	−0.10	0.12
b2	0.59	0.58	−0.56	1.73	2.39	0.73	0.93	3.85
Indirect effect a1b1	−0.69	0.20	−1.12	−0.35	−0.15	0.95	−2.24	1.49
Indirect effect a2b2	−0.07	0.08	−0.26	0.06	−1.16	0.56	−2.42	−0.25
* Indirect effect a1b1	−0.22	0.06	−0.34	−0.12	−0.02	0.15	−0.36	0.23
* Indirect effect a2b2	−0.02	0.03	−0.08	0.02	−0.18	0.08	−0.36	−0.04

Coefficient (unstandardized regression weights); SE (standard error); LLCI (lower bound of 95% confidence interval); ULCI (upper bound of 95% confidence interval). For all indirect effects bootstrapped standard errors, upper and lower bounds of 95% percentile confidence intervals are reported. * completely standardized specific indirect effect.
